# Impacts of stormwater pipe materials and pipe repairs on stormwater quality: a review

**DOI:** 10.1007/s11356-023-30508-6

**Published:** 2023-11-04

**Authors:** Mehwish Taneez, Heléne Österlund, Lian Lundy, Maria Viklander

**Affiliations:** Urban Water Engineering, Department of Civil, Environmental and Natural Resources Engineering, Luleå University of Technology, 971 87, Luleå, Sweden

**Keywords:** Stormwater, Pipe materials, CIPP, Metals, Organic contaminants

## Abstract

The water quality implications of transferring stormwater through pipes composed of concrete (new and used), polyvinyl chloride (PVC), galvanized corrugated steel (GCS), high-density polyethylene (HDPE), and pipes subjected to cured in place pipe (CIPP) and spray in place pipe (SIPP) trenchless repair technologies on stormwater quality are reviewed. Studies involve either the use of flowing water or an immersion experimental design, with data showing contact with pipe materials can affect stormwater quality parameters including pH, electrical conductivity (EC), and concentrations of minerals, metals, and organic constituents, e.g. styrene. ‘In-transport’ changes in pH (1–3 units), EC (2–3-fold), bicarbonate (3–44-fold), and calcium (2–17-fold) in stormwaters were reported following exposure to concrete pipes. Differences between the use of synthetic and field-collected stormwater were identified, e.g. turbidity levels in field-collected stormwater reduced on passage through all pipe types, compared to synthetic water where levels of turbidity on exposure to concrete and cement-based SIPP increased slightly. Transfer through PVC and HDPE pipes had minimal effects on physicochemical parameters, whereas exposure to galvanized corrugated steel pipes led to increases in EC, Zn, and Pb. Though limited data was available, the use of CIPP repairs and associated waste condensate generated during thermal curing and/or incomplete curing of resins was identified to release organic contaminants of concerns (e.g. styrene, vinylic monomers, dibutyl phthalate (DBP), diethyl phthalate (DEP), and benzaldehyde). The implications of findings for both future research and stakeholders with responsibility for reducing diffuse pollution loads to receiving waters are considered.

**Graphical Abstract:**

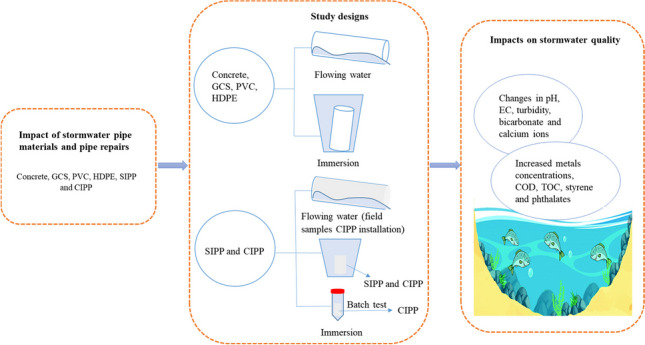

**Supplementary Information:**

The online version contains supplementary material available at 10.1007/s11356-023-30508-6.

## Introduction

Urbanization changes the natural hydrological cycle, with the main causative factors driving change being the widespread use of impermeable surfaces in conjunction with the artificial routing of water into subsurface drainage networks which directly discharge to receiving waters (Arnold and Gibbons 1996; Mcgrane 2016). These land use modifications can have significant impacts on the water environment, with increased volumes of urban stormwater runoff and flooding, in addition to degradation of receiving water quality (Marsalek et al. 2008). Urban stormwater runoff serves as a vector for the transport of a diverse range of pollutants (i.e. physical, chemical, and biological) from urban areas to receiving surface and/or groundwater water bodies, negatively impacting their quality (Goonetilleke et al. 2005; Müller et al. 2020). This diffuse form of pollution is identified as a leading cause of degraded receiving water quality and impaired ecological communities, with this collective environmental problem termed as the urban stream syndrome (Meyer et al. 2005).

The legislative requirements for reducing the discharge of, e.g. priority (hazardous) substance under the EU Water Framework Directive (WFD) require monitoring of receiving water quality and, where standards are not met, the development and implementation of pollution control strategies (i.e. Programmes of Measures) to enable the objective of ‘good status’ to be achieved (EU WFD 2000). One approach to mitigating diffuse pollution loads is to identify sources and limit activities leading to pollutant release if complete source elimination or substitution is not possible (Viklander and Marsalek 2011). Compared to other sources of stormwater pollutants, the stormwater pipe network itself has received relatively little attention in terms of its contribution to the in-transport pollution loads, and how the choice of stormwater pipe materials and pipe repairs technology may impact stormwater quality (Wright et al. 2011; Grella et al. 2016; Borris et al. 2017). Furthermore, stormwater contributes to concrete degradation through carrying aggressive solutes (acids, alkalis, sulphates, etc.) leading to decalcification, which causes leaching of calcium, bicarbonates, and other ions. This decalcification process reduces the mass, strength, and service life of the concrete pipe (Vipulanandan and Liu 2002).

An alternative pipe maintenance approach to pipe excavation and replacement is in situ maintenance using cured in place pipe (CIPP) repairs, a process involving the insertion of an uncured resin material within the damaged pipe. The uncured resin tube matrix consists of either a felt or fiberglass matting containing unsaturated resins (i.e. polyester, vinyl ester, and epoxy) and may be coated with, e.g. polypropylene, polyethylene, and/or polyvinylchloride. Polyester and vinyl ester resins contain styrene (up to 30–50% by weight) whilst epoxy resins do not. Other substances that can be present in a resin tube are monomers of, e.g. styrene; C_6_H_5_CH = CH_2_, bisphenol A diglycidyl ether; C_21_H_24_O_4_, vinyl ester; (RCOOCHCH_2_)n, initiators (e.g. Perkadox®; C_22_H_38_O_6_, Trigonox®; C_9_H_18_O_3_, Butanox®; C_8_H_18_O_6_, N,N-dimethylaniline; C_6_H_5_N(CH_3_)_2_), inhibitors (e.g. hydroquinone; C_6_H_6_O_2_), and fillers (talc; Mg_3_Si_4_O_10_(OH)_2_, silica; SiO_2_ crystalline or colloidal silica (amorphous), sodium metasilicate; Na_2_SiO_3_) (Tabor et al. 2014; Sendesi et al. 2020). Once in place, the pipe liners are then hardened using either thermal- (hot water or steam) or ultraviolet (UV) light-curing methods, with the duration of curing depending on the size of the pipe (from a few to several hours), followed by cutting off the closed end of the pipe liner to allow water to flow through the repaired pipe (Donaldson 2009; Sendesi et al. 2020). Some applications of CIPP also involve the use of synthetic liners (e.g. ultraliner and troliner) coupled with steam treatment or grouting to provide a watertight seal between the liner and the damaged pipe wall (Ren and Smith 2012). Another method of pipe repair is spray on liner or spray in place pipe (SIPP) liner in which a liner material is coated on the internal side of pipe using a spraying tool without using felt or fiberglass matting. Common lining materials include cement mortar, epoxy, polyurethane, and polyurea (Ellison et al. 2010; Whelton et al. 2013). CIPP and SIPP lining materials may also release substances, e.g. styrene, vinylic monomers, phthalates, and metals during the installation process itself (e.g. from the open-air handling of uncured resin, the improper disposal of CIPP condensate water produced during thermal curing, and/or the incomplete curing of resins) which can also increase alkalinity, total organic carbon (TOC), and chemical oxygen demand (COD) of contact waters.

Hence, both pipe materials and the application of CIPP/SIPP rehabilitation technologies have the potential to release a range of inorganic and organic contaminants into stormwater, with released substances and concentrations varying in relation to differences in pipe materials and runoff physicochemical characteristics. The EU WFD (EU WFD 2000) identifies the need to control diffuse pollution and the use of source control measures provides an opportunity to minimize contaminant release into the environment. Stormwater composition varies in relation to the land use type/activities (Müller et al. 2020), and its transfer to receiving waters typically involves transport through a pipe system. Hence, in addressing stormwater quality, it is also important to consider the impact of stormwater contact with pipe materials on the behaviour of stormwater contaminants. This article reviews the current scientific literature addressing the effects of stormwater pipes materials and the use of pipe lining technology on stormwater quality and changes in physicochemical parameters due to experimental methodology and discusses the implication of findings for stormwater management.

## Methodology

A search of the Scopus and Web of Science databases was undertaken using the following key terms: pipes and pipe linings and pipe repairs, stormwater quality, and metals transport in pipes. This returned 478 papers, which were further evaluated to identify articles which focused on the effects of various pipe materials on stormwater quality through use of the following search strings: ‘stormwater and pipe materials’ and ‘stormwater and pipe repairs’. This led to a ‘long list’ of 204 articles. Google Scholar was also used to identify further relevant articles (*n* = 2) not captured by the selected databases. The 204 articles were then manually reviewed to identify those which specifically refer to water quality changes following contact with pipe materials or pipe repairs either in the field or laboratory. This led to the identification of 13 articles for consideration in this review.

## Results and discussion

### Pipe materials and experimental conditions used in previous studies

The details of each study (i.e. pipe materials, pipe repair technology, and parameters analysed) are provided in the supplementary data (Table S1). In-transport changes of stormwater on passage through pipes were reported in seven articles and focused on changes associated with exposure of water to concrete pipes (unused or immediately on installation referred to henceforth as ‘new’ and/or old (i.e. field deployed), steel reinforced and fiber reinforced), polyvinyl chloride (PVC), high-density polyethylene (HDPE), and galvanized corrugated steel (GCS). In addition, the effects of pipe liners repair technologies (SIPP and CIPP) were evaluated in six articles in terms of pollutant emissions.

**Fig. 1:**
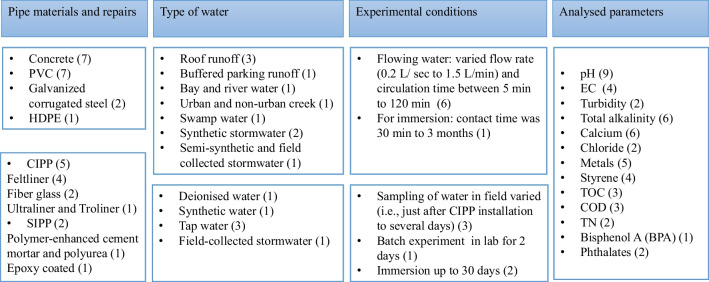
Overview of experimental design used in identified studies. Key: number in brackets refers to the number of times the parameter/condition is included in an article

An overview of the experimental designs used in the short-listed articles is summarized in Fig. 1, and identifies key variations in (i) sections of different pipe materials and pipe repair (SIPP/CIPP) utilized, (ii) water type utilized, (iii) flow/immersion of pipe section conditions, and (iv) physiochemical parameters determined in pre- and post-circulated water. The details of physiochemical parameters reported in each study are given in Table 1.

**Table 1:** Overview of physicochemical parameters analysed per study

Study	pH	EC	Bicarbonate	Ca	Turbidity	Metals	Organics
Perkins et al. 2005						×	
Davies et al. 2010b	×	×	×				
Davies et al. 2010c	×	×	×				
Ogburn et al. 2013	× *	× *	× *	× *		×	
Grella et al. 2016	×	×	×				
Borris et al. 2017	×				×	×	
Purdy et al. 2021	×	×		×		×	
Donaldson 2009							×
Ren and Smith 2012							×
Whelton et al. 2013	×		×		×		×
Donaldson and Whelton 2013	×						×
Tabor et al. 2014				×		×	×
Li et al. 2019	×						×

*Data taken from PhD dissertation (Ogburn 2013)

### Changes in water quality parameters on exposure to pipe materials and pipe repair technologies

#### Impact on pH levels

**Fig. 2:**
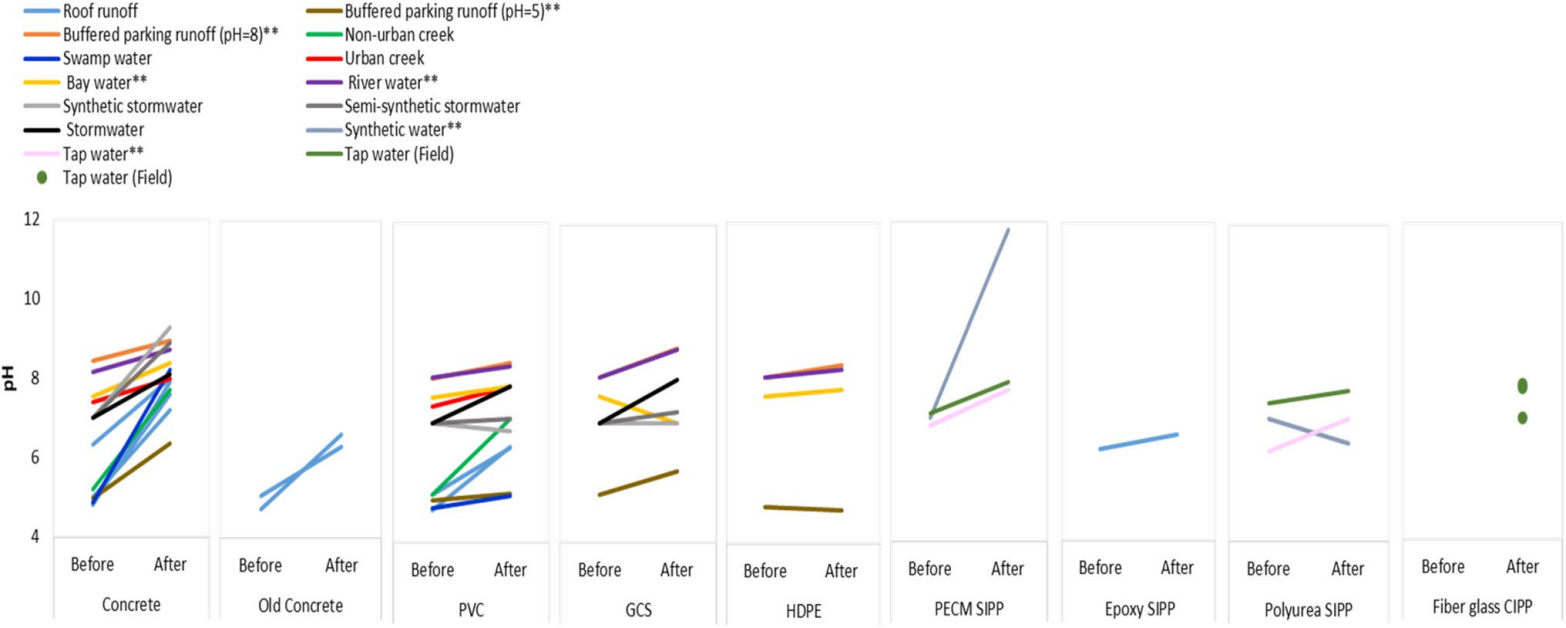
pH changes in contact waters exposed to pipe materials in flowing and immersion (double asterisks) studies, data adapted from Davies et al. (2010b, c), Ogburn et al. (2013), Donaldson and Whelton (2013), Whelton et al. (2013), Grella et al. (2016), Borris et al. (2017), Li et al. (2019), and Purdy et al. (2021). Key: PVC, polyvinyl chloride; GCS, galvanized corrugated steel; HDPE, high-density polyethylene; PECM, polymer-enhanced cement mortar; SIPP, spray in place pipe; and CIPP, cured in place pipe

Studies involving exposure of stormwater to different types of pipe materials revealed that irrespective of pipe material and starting pH (which varied from pH 4.7–8.4), the pH increased in the majority of studies (minor decreases noted for PVC and polyurea SIPP (synthetic water), HDPE (buffered water pH = 5), and GCS (bay water)), with exposure to concrete pipes having the greatest impact (Fig. 2). The effect of new concrete pipes on pH was greater (a mean increase of 2.4 ± 0.6 pH units) compared to exposure to aged concrete (age estimated to be at least ⁓75 years; a mean increase of 1.5 ± 0.4 pH units). The increase of pH associated with exposure to new concrete pipes was as a result of water reacting with cement components (e.g. tri-and di-calcium silicates) to form hydration products such as calcium silicate hydrate and calcium hydroxide (Davies et al. 2010b).

The pH of stormwater is also reported to change on contact with lining materials used in SIPP and CIPP repair technologies. Whilst only limited data is available (see Table 1, *n* = 4), for example, exposure to an epoxy-treated concrete pipe led to an increase of 0.37 pH units. However, this was less than that observed for exposure to untreated concrete (pH increase of 1.31 units). Minor changes in pH value were observed due to the presence of liners, e.g. for epoxy (from 6.32 to 6.69) and polyurea (from 7.5 to 7.8), with the authors suggesting that flowing water led to increased levels of aeration and the release of carbon dioxide from solution and subsequent shifting of pH (Grella et al. 2016). However, experimental conditions (i.e. flowing water vs immersion; type of water used) may also result in different end pH values. For example, 3 days of exposure to a polymer-enhanced cement mortar liner (PECM-SIPP) (immersed in synthetic water) led to an increase in pH from 7.1 to 11.8. In comparison, the immersion in tap water led to an increase of pH from 6.9 to 7.2, with a change from pH 7.2 to 8 reported when tap water was flowing. The greatest increase in pH was measured in an experiment involving the immersion of PECM-SIPP in synthetic water where, after 3 days, the pH increased from 7.1 to 11.8. In contrast, exposure of synthetic water to a polyurea liner led to a decrease in pH from a mean value of 7.1 to 5.9, with this pH reduction understood to be a function of unreacted isocyanate resin reacting with water which yielded primary amines (RNH_2_) and carbon dioxide (Donaldson and Whelton 2013). Regarding field studies, only Li et al. (2019) reported the pH values in flowing water (tap water; initial pH not given) after field installation of UV cured CIPP (fiberglass) at four sites as 7.95, 7.85, 7.92, and 7.09 (Fig. 2). However, as initial pH values were not reported, it is not possible to assess the impact of exposure to this repair technology on pH.

Exposure of contact waters to PVC pipes leads to an increase in pH (from 0.1 to 1.9 pH units), to galvanized corrugated steel (from 0.3 to 1.1 pH units), and to HDPE (from 0.1 to 0.3 pH units) (Ogburn et al. 2013; Borris et al. 2017). The pH increase in contact waters exposed to PVC was greatest for non-urban creek and roof runoff waters (initial pH 4.8–5.2; increase of up to 1.9 pH units after circulation), whilst the change in pH was < 1 pH unit for all other water types. pH changes in synthetic or semi-synthetic stormwaters were minimal on contact with GCS, with only a minor increase in pH (0.5–1.1 units) observed on exposure of field-collected stormwater, buffered parking lot runoff, and river water to the same material (Ogburn et al. 2013; Borris et al. 2017).

Data indicates that PVC pipe materials generally have relatively smaller changes in the pH of flowing waters, except for non-urban creek and roof runoff waters (where larger pH increases up to 1.9 units were reported) which was associated with its relatively low levels of alkalinity (0.5–1.7 mg/L). However, studies have also shown that exposure to PVC materials may have an acidifying effect (i.e. a lowering of pH was observed in synthetic waters by Borris et al. 2017 and De Buyck et al. 2021) which they associated with the release of acids on PVC degradation. An alternative explanation is the decrease in pH may be due to the leaching of phthalic acid esters (used as plasticizers) and fatty acids (lubricants) (Story et al. 2000). The potential acidifying effect of PVC materials suggests that even ‘inert’ materials can impact on water quality and highlights the need for long-term material evaluation in diverse environmental conditions.

Overall, whilst the impact of stormwater pipe materials on the pH of contact waters varied in relation to both pipe material and the type of water utilized, the pH associated with field-collected stormwater typically showed an increase irrespective of pipe material. However, the increase of pH was most prominent when mineral-poor water (i.e. low alkalinity) (e.g. rainwater, synthetic stormwater, swamp, and non-urban creek) was circulated, compared to the use of field-collected stormwater, river water, bay water, and water from urban creeks. Reported pH increases can be up to several pH units, with the dissolution of calcareous material from concrete pipes largely a function of the pH of the water which comes in contact with these surfaces (Davies et al. 2010c; Ogburn et al. 2013; Borris et al. 2017). Natural streams (i.e. < 5.0% catchment imperviousness) were found to be acidic in comparison to urban streams (neutral to slightly alkaline with high electrical conductivity) (Wright et al. 2011; Tippler et al. 2012).

The complex relationship between pipe materials and stormwater types described here emphasizes the need for careful selection of pipe materials, especially in the areas with varying water characteristics. HDPE pipes may be more suitable for some contexts due to their limited impact on pH, whilst the use of concrete pipes may require additional consideration and mitigation measures. Variations in stormwater pH can also impact downstream water treatment processes, necessitating adjustments in treatment methods based on the materials used in stormwater systems.

#### Changes in electrical conductivity (EC), bicarbonate, and calcium

Increases in EC values were reported for all contact waters on exposure to concrete pipes (Figure S1 in the supplementary data). For example, increases in EC on exposure to concrete pipes varied with pipe age, from up to a 3.9-fold increase on exposure to new concrete to a 1.4-fold increase for an old concrete pipe (~ 75 years) and a 1.0-fold increase on exposure to an epoxy-lined pipe (same water types). In relation to contact water type, the levels of EC increase in buffered parking lot runoff, urban creek, and bay water were relatively lower (0.9–1.2-fold) compared to roof and swamp waters where EC levels doubled after flowing through concrete pipes due to low buffering capacity of these waters. Compared to exposure to concrete, the EC increases associated with exposure to PVC (0.99–1.5-fold), galvanized corrugated steel (0.84–1.5), and HDPE (1.3–1.4) pipe materials were relatively lower and of a similar magnitude of change (Davies et al. 2010c; Ogburn et al. 2013; Purdy et al. 2021). As natural streams (i.e. < 5.0% of catchment imperviousness) are relatively acidic with typically low levels of EC, the use of concrete pipes as a part of urban drainage systems can lead to rapid changes in the EC of contact waters — and potentially receiving waters — compared to scenarios involving the use of other materials (e.g. PVC, HDPE, and galvanized corrugated steel) in stormwater pipelines. In contrast, the use of an epoxy liner (applied via either CIPP or SIPP) is shown to limit the increases in EC compared to changes associated with exposure to non-treated concrete (Grella et al. 2016). However, this relates to a single study and no further laboratory or field data on the effects of the SIPP and CIPP processes on EC levels associated with pipes of other material types could be sourced.

The leaching of substances from concrete pipes provides an artificial source of ionic compounds (particularly calcium and bicarbonate ions) into urban waterways, with data indicating that the level of leaching was affected by the contact water type and concrete pipe age (Davies et al. 2010b; Grella et al. 2016; Purdy et al. 2021). For example, the transport of roof runoff water through new concrete pipes increased calcium (from 0.5 to 7.0 mg/L) and bicarbonate concentrations from 0.5 to 22 mg/L, respectively, compared to exposure to older concrete materials (calcium; from 0.5 to 2 mg/L, bicarbonate 0.5 to 5.3 mg/L). Aggressive waters (i.e. with a pH ≤ 5) dissolve more ions from a concrete surface compared to buffered waters with an initially higher bicarbonate concentration (Davies et al. 2010b, c). The application of liners or coatings within concrete pipes (i.e. use of CIPP) can limit the dissolution process with data indicating that bicarbonate, Ca, Na, and K concentrations in rainwater were unaffected following exposure to pipes that had undergone this repair process (Grella et al. 2016). However, an increase in Ca concentration (from 12.5 to 63.8 mg/L) was reported in receiving waters immediately after field installation of steam-cured CIPP, with this increase reported to be due to the presence of calcium in the CIPP condensate. A further study also reported a (lower) increase in Ca concentration after 7 days of CIPP installation of 23.4 to 29.6 mg/L (Tabor et al. 2014) and hence further research is required to enable a full understanding of processes and effects to be developed. Whilst guidelines for CIPP installation (including curing processes) exist, it is essential to also introduce compliance checks (i.e. to assess whether they are consistently followed) to safeguard water quality in urban environments. In terms of overall impacts, the data indicates that the buffering capacity of contact waters plays a crucial role in determining the magnitude of EC increases. Waters with low buffering capacity are identified as more susceptible to significant changes in EC, potentially leading to adverse effects on aquatic ecosystems.

#### Impact on levels of turbidity

Studies in the literature have shown that pipe materials and pipe repairs may induce changes in the levels of turbidity of transported stormwater. For example, Borris et al. (2017) explored the impact on levels of turbidity in synthetic, semi-synthetic, and field-collected stormwater flowing through sections of concrete, PVC, and galvanized corrugated steel pipes. A minor increase in synthetic stormwater turbidity (from 1.4 to 3.5 NTU) occurred on exposure to concrete, with this identified as due to particle detachment from the concrete pipe surface. In contrast, levels of turbidity in synthetic stormwater were unaffected by exposure to either PVC or galvanized corrugated steel pipes, although whether this was a function of the pipe materials (PVC and galvanized corrugated steel), their smooth surfaces (no detachment of particles was observed) or a function of synthetic stormwater composition was not clear. For instance, both synthetic stormwater studies did not include sediment particles and so direct comparisons of studies are challenging. Semi-synthetic and field-collected stormwater turbidities were reduced after flowing through PVC (14–16%), concrete (42–50%), and galvanized corrugated steel pipes (67–85%). Decreases in turbidity were associated with the physical detention of particles, e.g. within pipe corrugations (a function of pipe design) or surface roughness, combined with cement-induced flocculation of particles (Figure S2 in the supplementary data). This minor variation in turbidity reduction between two types of waters for the pipe materials in this study is associated with the fact that both water types reported similar total suspended solids concentration (150 mg/L).

Data generated through immersion experiments with polyurea and PECM-SIPP indicated a 4.2-fold increase in turbidity of synthetic waters, whilst exposure of the same type of contact water to polyurea-SIPP increased turbidity by 2.8-fold (Whelton et al. 2013). The increase in turbidity of synthetic water was due to the nature of SIPP materials (cement based and polyurea) — which release particles into water. However, no data is available on turbidity of water after CIPP field installation or in laboratory flowing water studies. Given the lack of data on turbidity after CIPP installations, further research is needed to assess the impact of pipe repair methods on stormwater turbidity. Considering the composition of stormwater, the choice of pipe materials is a critical factor that can impact turbidity levels over time. For example, new corrugated galvanized steel pipes can trap particles within corrugates. Later, during heavy rainfall events or high flows, these trapped sediments may potentially be resuspended and flushed out, leading to changes in the turbidity levels of stormwater. Similarly, concrete pipes over time may also contribute to increases in turbidity due to particle detachment. Understanding the mechanisms behind turbidity reduction, such as physical particle detention and flocculation and stormwater composition, can help in designing more effective stormwater management systems, allowing for better control of turbidity levels.

#### Impact on metals concentrations and speciation

**Fig. 3:**
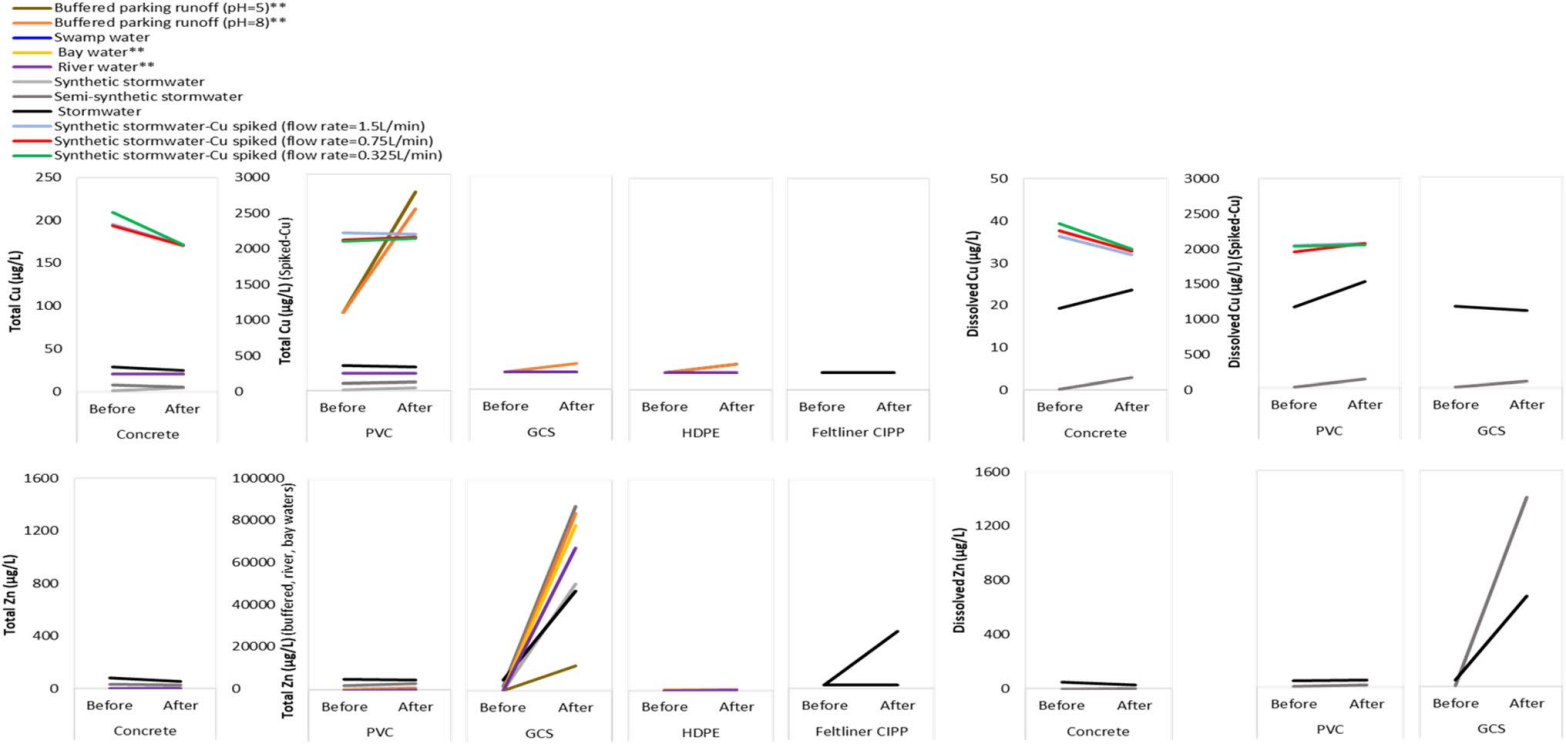
Changes in total and dissolved Cu and Zn concentrations in contact waters in flowing and immersion (double asterisks) studies due to pipe materials, data adapted from Perkins et al. (2005), Ogburn et al. (2013), and Borris et al. (2017). Key: PVC, polyvinyl chloride; GCS, galvanized corrugated steel; HDPE, high-density polyethylene; and CIPP, cured in place pipe

Metals in urban stormwater runoff are arising directly from atmospheric deposition as well as due to the mobilization from urban dusts and leaching from exposed materials on contact with rainfall (Müller et al. 2020). Studies showed that both the concentration of metals and their speciation within contact waters may change on exposure to pipe materials (Perkins et al. 2005; Borris et al. 2017). For example, total Zn concentrations associated with exposure to galvanized corrugated steel pipes were higher compared to exposure to other pipe materials for all water types (see Fig. 3). Zn increases up to several orders of magnitude in different waters were observed, e.g. from − 4 to 815 µg/L (synthetic stormwater), 32.1 to 1406 µg/L (semi-synthetic), 81.1 to 759 µg/L (stormwater), 20 to 11,700 µg/L (buffered water pH = 5), 20 to 84,300 µg/L (buffered water pH = 8), 20 to 78,600 µg/L (bay water), and 20 to 67,800 µg/L (river water) (Ogburn et al. 2013; Borris et al. 2017). Among all the water types used, buffered parking runoff (pH = 8) showed maximum leaching of Zn from galvanized corrugated steel pipes followed by bay and river water (pH = 8) which could be due to the amphoteric properties of Zn and the formation of divalent zincate-anion (CaZnO_2_) or sodium zincate (Na_2_[Zn(OH)_4_]) which respond differentially to differences in composition of waters used and immersion time periods. Galvanized steel is susceptible to corrosion by water containing excess free carbon dioxide and waters with a pH that is > 8.2 lead to the release of dissolved Zn into solution (Brandt et al. 2017). Similarly, the varying composition in contact water type is believed to be the contributing factor to the differing Zn concentration observed in the tested waters, synthetic stormwater > semi-synthetic stormwater > field-collected stormwater (Borris et al. 2017).

With regard to other pipe materials, Zn concentrations in the circulated waters were relatively lower on exposure to concrete pipes (except for buffered parking runoff (pH = 8) and synthetic stormwater where slight increases of 20 to 30 µg/L and < 4 to 5.53 µg/L, respectively, were reported). The leaching pattern of Zn for buffered waters was PVC > HDPE > concrete, except for bay and river waters which showed no change in Zn concentrations for the same pipe materials. Exposure to CIPP liners may also lead to elevated Zn concentrations in flowing waters, with a field inspection post-CIPP installations to repair damaged pipes showing differences in Zn concentrations at upstream and downstream sites. Specifically, the concentration at the repaired pipe outlet increased immediately after the installation process (from < 30 to 440 µg/L) with the source of Zn identified as a polymer matrix reinforcement additive detected within CIPP condensate (cure water) generated during thermal curing of CIPP resin (Tabor et al. 2014).

Limited data is available for comparison on changes in dissolved Zn concentrations in differing types of waters during transport through pipe materials. Borris et al. (2017) reported changes in dissolved Zn concentration in semi-synthetic and field-collected stormwater on contact with pipe materials, indicating that galvanized steel pipes were a larger source of Zn (with Zn mainly present in the dissolved form), in comparison to exposure to concrete and PVC pipes. In semi-synthetic stormwater, Zn concentrations increased by 1400 µg/L upon contact with galvanized steel pipes, whilst in field-collected stormwater, the increase was 670 µg/L indicating that difference in metal concentration and speciation due to variation in water composition may have consequences for water quality and the surrounding ecosystem. The observed differences between studies suggest the need for further investigation into the complex relationship between water composition and the release of metals from pipe materials, and if certain types of waters are more likely to cause increased metals release from pipes, it could contribute to the development of targeted mitigation approaches for the protection of water resources and human health.

Changes in total Cu concentrations in contact waters exposed to different pipe materials (i.e. concrete, PVC, galvanized corrugated steel, HDPE) were relatively minor irrespective of the water type used (Perkins et al. 2005; Ogburn et al. 2013; Borris et al. 2017) (see Fig. 3). Moreover, field stormwater samples did not show any change in total Cu (or Pb) concentration due to CIPP material (Tabor et al. 2014). However, an increase in total Cu concentration from 90 to 230 µg/L was observed in buffered (to pH 5) parking lot runoff exposed to PVC pipes for a period of 3 months, indicating that acidic conditions may increase leaching of metals (Ogburn et al. 2013).

Furthermore, an increase in dissolved concentration of Cu in Cu-spiked synthetic stormwater (from 1947–2040 to 2043–2070 µg/L), semi-synthetic (from 0.21 to 2.02 µg/L), and field-derived stormwater (from 19.3 to 25.3 µg/L) was observed on passage through PVC pipes (Perkins et al. 2005; Borris et al. 2017). This increase in dissolved concentration after contact with PVC may be due to speciation changes (i.e. from particulate-associated to a dissolved form) because the total Cu concentrations were less affected on exposure to PVC (Perkins et al. 2005; Borris et al. 2017). Dissolved Cu increased in all waters exposed to concrete pipes, except for Cu-spiked synthetic water where it decreased by 13–18%. This may be due to adsorption of dissolved Cu ions onto the concrete pipe surface from synthetic water (Perkins et al. 2005). Borris et al. (2017) reported an increase in dissolved Cu concentrations in contact waters on exposure to concrete, i.e. in semi-synthetic stormwater from 0.21 to 2.84 µg/L and in field-collected stormwater from 19.3 to 23.6 µg/L. The difference in behaviour of Cu-spiked synthetic and field-collected stormwater indicates that in field environments Cu may not be adsorbed after passing through concrete and questions the representativeness of using synthetic stormwater in environmental behaviour and fate studies. The observed variations in Cu concentration and distribution (between different phases) in different stormwater types on exposure to pipe materials also serve as an indicator that environmental systems are intrinsically complex. Additionally, factors such as pipe age and pH may influence the release, adsorption, and transformation of metals during transfer through pipe materials.

The total Pb concentration in semi-synthetic and field-collected stormwater decreased on exposure to all pipe materials in both flowing and immersion water design experiments (except for buffered parking lot runoff for galvanized corrugated steel see Figure S3 in supplementary information) whilst for synthetic stormwater, bay, and river waters total Pb concentration was below the detection limit before and after exposure to all pipe materials. The decrease in total Pb concentrations in semi-synthetic stormwater and field-collected stormwater on exposure to PVC was 53–71%, concrete (78–83%) and galvanized corrugated steel (91–87%) (Borris et al. 2017). In an immersion test (duration ranges from 0.01 h to 3 months), no difference in total Pb concentrations after 1 day exposure was observed for all pipe materials in all water types, with a limited decrease in total Pb concentrations observed after 3 months, i.e. PVC ≈ HDPE (15–18%) ˃ concrete (5–11%) > galvanized corrugated steel pipes (9%) (Ogburn 2013; Ogburn et al. 2013). The difference in results between the above two studies may arise due to difference in methodology used (i.e. flowing water vs immersion). The results from the long-term immersion of galvanized corrugated steel pipes in buffered parking runoff (pH = 5 and 8; initial Pb concentration of < 5 µg/L) led to an increase in Pb concentration (up to 247 µg/L and 628 µg/L), respectively. The leaching of Pb is favoured under acidic pH conditions, with the release of Pb under alkaline pH (i.e. buffered water pH 8) being associated with its amphoteric character, i.e. ability to react with both acids (formation of salts) and bases (formation of soluble hydroxyl complexes) (Ogburn et al. 2013; Król et al. 2020). The source of Pb in the galvanized steel pipes is understood to be its co-occurrence with Zn (commonly smelted Zn contains <1 Pb %) (Wasserstrom et al. 2017). In contrast, synthetic stormwater did not result in the elution of Pb from concrete, galvanized corrugated steel, or PVC, with a decrease from 0.05 to 0.02 µg/L reported on the exposure of semi-synthetic stormwater. The exposure of field stormwater to pipe materials induced minor increases in dissolved Pb content from 0.12 to 0.16 µg/L (concrete), 0.18 µg/L (galvanized corrugated steel), and 0.24 µg/L (PVC) (Figure S3) (Borris et al. 2017). A possible explanation for the observed increase on exposure to PVC (from 0.12 to 0.24 µg/L) could be due to the use of Pb compounds, 0.5–3%w/w as thermal stabilizers (Hahladakis et al. 2018). A study by Purdy et al. (2021) observed that the exposure of swamp water to concrete pipes can elevate the concentrations of several elements, including Cr, Ca, K, and Sr. The largest increases were recorded for Sr (from a pre-circulation concentration of < 1 µg/L to a post-circulation concentration of 13.9 1 µg/L).

The implications of metals release from pipe materials in stormwater are multifaceted and have significant consequences for water quality. Key findings suggest that water composition, pH, and speciation play critical roles in influencing metal releases from various pipe materials. Moreover, the magnitude of metals release from same pipe material (e.g. GS) may vary depending on the specific compositions of water and the aging of the pipes, leading to the discharge of stormwater with varying metal concentrations into receiving waters. Concrete pipes are identified as a potential source of various metals in runoff, with the potential to disturb the equilibrium of aquatic ecosystems. However, the release of metals was found to be lower from pipe repairs (except for Ca and Zn) although only limited data is available. The leaching of metals from pipe repairs may depend on the type of resin curing technology adopted, the extent to which resin is cured and site conditions (e.g. continuous or intermittent water flow). Further research on pipe repairs is needed to explore the effects of complete and incomplete curing on metals release under field conditions.

#### Organic contaminants leaching from stormwater pipe repairs

In CIPP applications, three types of unsaturated resins — polyester (OCR_1_COOR_2_O)n, vinyl ester (a hybrid of vinyl ester and epoxy; (RCOOCHCH_2_)n), and epoxy (C_21_H_25_ClO_5_) infused felt- or glass-liners — are commonly used. The use of CIPP can lead to the release of organic substances into the surrounding environment both during and post-CIPP installation processes, including styrene (C_6_H_5_CH = CH_2_) which is present in the first two resins (30–50% by weight) (Donaldson 2009). For examples, the monitoring of seven stream sites in Piedmont and Blue Ridge provinces of Virginia, USA (see Table S1), during and after the application of steam-cured CIPP installation processes, indicated that styrene occurred in receiving waters at a range of concentrations for varying durations reflecting differences between sites (e.g. water flow volume and rate, and differences in handling of installation waste materials). For example, steam condensate with a styrene concentration of 77 mg/L was reported to have been discharged to downstream waters during installation, with styrene concentrations downstream of discharge points exceeding both Canadian water quality guidelines for the protection of aquatic life (CCME 1999) and aquatic toxicity thresholds established for *Daphnia magna* and *Pimephales promelas* (Cushman et al. 1997; Tabor et al. 2014). Styrene was detected up to 88 days following discharge (study period from day 0 to day 120), with higher concentrations at sites with intermittent water flow suggesting that flow characteristics influenced levels of dilution. However, styrene was also detected downstream of sites where condensate was not discharged to downstream, with sources suggested to include the incomplete curing of resin-saturated liner (Donaldson 2009).

In terms of other substances, analysis of uncured resin showed the presence of more than 70 compounds including bisphenol A diglycidyl ether (BADGE) (1110 ± 40 mg/kg), 1-hydroxycyclohexyl phenyl ketone (2270 ± 80 mg/kg), dibutyl phthalate (DBP) (388 ± 60 mg/kg), and benzaldehyde (130 ± 11 mg/kg) (Li et al. 2019). Other contaminants detected in CIPP materials and/or waste streams include benzene, diethyl phthalate (DEP), DBP, benzyl alcohol, 4-(1,1-dimethyl) cyclohexanol, and 4-(1,1-dimethyl) cyclohexanone (Tabor et al. 2014). This a concern as several of these substances, e.g. DBP, DEP, and BADGE are classified as suspected endocrine disrupters (Tabor et al. 2014; Li et al. 2019).

The potential for organic contaminant leaching during CIPP installations varies with the curing method employed. For example, UV-curing methods for CIPP installations do not produce steam condensate, a factor used to explain why its use was associated with an over 10-fold lower receiving water styrene concentration (0.45 mg/L) in a field study in comparison to the use of steam-cured CIPP when styrene concentrations of 5.48 mg/L were detected (see Table S1). However, whether this is a function of the efficacy of the curing process or the absence of condensates generated with UV curing is unclear.

Differences in the concentrations of various contaminants leaching after UV-CIPP installations are also reported, e.g. styrene concentrations ranging from 3.3 to 446 µg/L, benzaldehyde from 12.5 to 68 µg/L, DBP from 6.3 to 12.5 µg/L, and 1-hydroxycyclohexyl phenyl ketone from BDL to 55.2 µg/L. Factors contributing to these reported ranges in pollutant release include differences in the composition of CIPP materials (often commercially protected) and the experimental methods used to derive data (i.e. flowing vs immersion methodologies) (Donaldson and Whelton 2013). A list of substances detected in CIPP resin, condensate, and in receiving water samples collected at CIPP installation sites is presented in Table 2.

**Table 2:** List of substances reported in CIPP resins and installation sites

Substances	Media where substance was found		
	Uncured CIPP resin	CIPP condensate	Stormwater sample
Benzene*		×	×
Benzaldehyde*¤	×	×	×
Benzyle alcohol*		×	×
Dibutyl phthalate (DBP)*¤	×	×	×
Diethyl phthalate (DEP)*¤	×	×	×
Styrene*¤ф	×	×	×
Vinylic monomer			×
1,2,4-Trimethyl benzene*¤	×	×	×
1,2,5-Trimethyl benzene*	×	×	×

*Tabor et al. (2014), ¤Li et al. (2019), фDonaldson (2009),  Donaldson and Whelton (2013)

The impact of using styrene-free resins in CIPP applications has also been explored. For example, in a study of the impact of vinyl ester-based UV-CIPP installations, vinyl and acrylate monomers were detected in receiving waters post-CIPP installation at concentrations of 76 mg/L and 0.009 mg/L, respectively, concentrations which exceed toxicity thresholds for fish and water fleas (Donaldson and Whelton 2013). In contrast, a laboratory-based batch test study of commercially available CIPP products ultraliner™ and troliner™ did not detect the targeted analytes bisphenol A (BPA), di-(2-ethylhexyl) phthalate (DEHP), and benzyl butyl phthalate (BBP) at concentrations above their reported levels of detection (LOD; LOD for BPA, DEHP, and BBP of 0.029 mg/L, 0.191 mg/L, and 0.447 mg/L, respectively) (Ren and Smith 2012).

With regard to the use of SIPP installations, a study by Donaldson and Whelton (2013) reported that concentrations of total organic carbon (TOC), chemical oxygen demand (COD), and total nitrogen (TN) in tap water were not affected by exposure to polyurea and PECM SIPP during open-air immersion or flowing water experiments (with the exception of TOC which increased from < LOD to 5.7 mg/L in flowing water post installation). In contrast, the immersion of polyurea SIPP in synthetic water (pH 7.1, 47 mg/L CaCO_3_) in sealed containers led to an increase in COD (from < LOD to 98.3 mg/L), TOC (from < LOD to 19.9 mg/L), and TN (from < LOD to 2.8 mg/L). However, the factors underpinning these different results cannot be elucidated from this study as different water types (selected to explore impact of different alkalinities) were used (Donaldson and Whelton 2013).

Studies on the release and impact of organic substances from pipe repairs are limited. However, a commonality in studies to date is that results vary in relation to the type of — and approach to — installation processes, i.e. poor handling of uncured resins, type of/incomplete resin curing, and/or management of steam condensate (produced as a result of thermal curing only) (Donaldson 2009). However, due to a lack of field studies, the extent to which identified challenges can be managed by the selection of curing approach and ‘good housekeeping’ measures in the field is not yet clear. Further research is therefore recommended, with a focus on the impact of and extent to which best practice guidelines for the installation of pipe repair technologies (e.g. NASSCO guidance to support the safe handling of resin materials) are implemented and complied with.

### Differences in physicochemical parameters due to experimental design

**Fig. 4:**
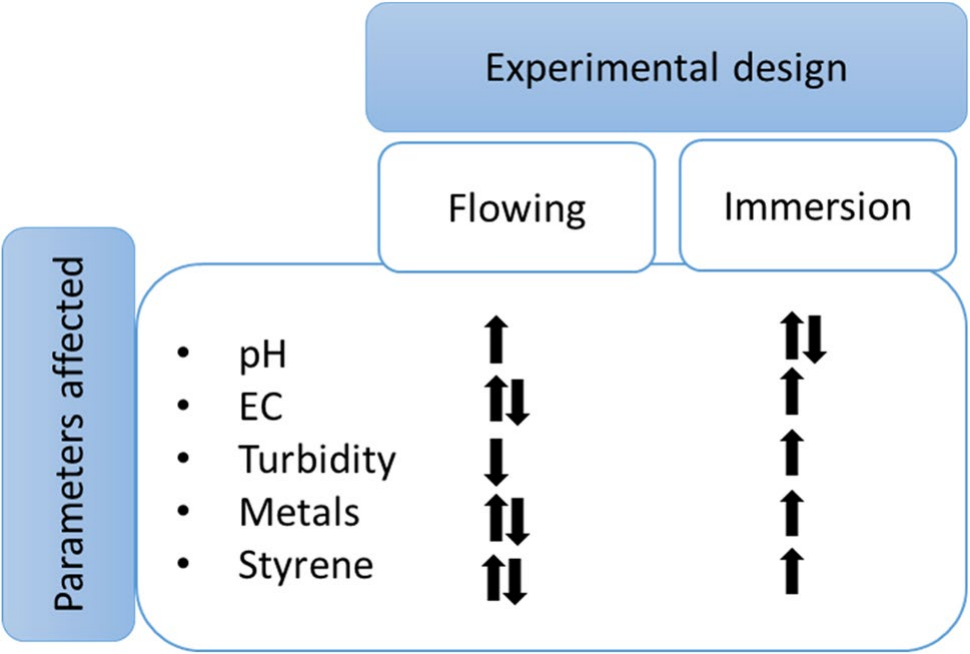
Overview of increases (identified as an upwards arrow) and decreases (identified as a downwards arrow) in concentrations of measured parameters associated with experimental design; having both upwards and downwards arrows indicates both increases and decreases reported

Changes in physicochemical parameters described above were found to be associated with both the pipe material and the composition of contact waters used. However, despite having difference in contact time in experimental design (immersion; max: 3 months, flowing water; max: 120 min), it highlights that some differences in parameters are through to be a function of relation to experimental design used (i.e. use of flowing waters vs immersion) and pipe material dependent (Fig. 4). For example, the results for turbidity, EC, and leaching of various substances (e.g. metals, styrene) in flowing water tests differed from those generated using immersion tests (when all other study aspects consistent). A pertinent example is the decrease in turbidity values reported in the flowing water design test was not observed in immersion test, with this difference associated with particle entrapment in the cavities and corrugates present in the concrete and galvanized corrugated steel pipes as water flowed through the pipes. As water did not move within the immersion test, the same processes did not occur, and turbidity did not decrease (Figure S2). Whilst these results could be used to indicate the strength of using a flowing water experimental test design as being more relevant to field conditions, it is also noted that under field conditions such cavities may fill quickly and no further entrapment of particles from stormwater may occur which may result in elevated turbidity values in the receiving water bodies if particles trapped earlier are mobilized during later events. In contrast, data indicates that EC may increase (1.5–2-fold) in waters exposed to, e.g. PVC and galvanized corrugated steel following long-term (3 months) immersion, with lower levels of increase reported for flowing water design tests, where the level of EC increase was relatively lower (by 1-fold). However, no flowing experimental data (> 120 min contact time) is available for PVC and galvanized corrugated steel pipes for comparison, and it can be of interest to determine whether the changes observed due to increased contact time in the immersion test could have similar trends in stormwater quality due to pipe age in flowing tests. Similarly, immersion impacted the leaching of metals from pipe materials, e.g. galvanized steel pipes tend to release more Pb and Zn into contact waters and Cu and Zn from PVC in buffered parking lot runoff as compared to their concentrations reported in flowing water tests (all other study aspects the same). In the case of styrene, the difference in experimental methodologies affected the level of styrene in contact waters. Styrene leaching from pipe liners increased in the immersion experimental design. This is thought to be due to its accumulation over time as compared to flowing water design experiments where styrene decreased gradually, as residual contaminants released from the liner did not have the same opportunity to accumulate. Although the use of immersion experimental design methods over extended time periods can support assessments of the long-term behaviour of pipe materials on stormwater quality, in the field, contact waters are unlikely to be stationary in stormwater pipes for such a long period. The flowing water experimental method in the laboratory studies may be more representative of field conditions than the immersion method. An exception to this, however, is a scenario where low to no-flow conditions (i.e. seasonal streams) leads to the ‘ponding’ of stormwater followed by a flush and under these circumstances an immersion test methodology may be more appropriate.

## Conclusions and environmental implications

Pipe systems play a major role in the conveyance of stormwater from urban areas to receiving waters. These conduits can be made up of different materials and, in addition, may have been subjected to one or more types of pipe rehabilitation technologies. Whilst the number of studies is limited, there is clear evidence that the type of materials/repair processes utilized can impact on transported water quality and that impacts are not consistent between water quality parameters, type of study water, pipe material, repair technology, and by experimental method employed. However, despite differences in study experimental design, available findings clearly indicate that the use of galvanized corrugated steel and concrete pipes and CIPP-lining residues (derived from the use of thermal curing), steam condensate, and extruded resin can act as a source of contaminants in stormwater. Concrete pipes, regardless of age, continue to leach bicarbonate and Ca ions to contact waters. This release of ions may change runoff — and ultimately receiving water quality, exerting pressure on aquatic biota, e.g. reduced availability of suitable habitats and/or food sources and distribution of benthic diatoms (Potapova and Charles 2003; Davies et al. 2010a; Wright et al. 2011; Tippler et al. 2012).

In addition, contact with certain pipe materials is reported to change total and dissolved metal concentrations during the transport process, with this effect most prominent on exposure to galvanized corrugated steel pipes. Zn releases from galvanized corrugated steel pipes were 2–4 orders of magnitude (from starting concentrations) to all types of water, as well as in comparison to the level of Zn release from other materials (i.e. PVC and HDPE in buffered waters only), with reported concentrations frequently exceeding the suggested guideline concentration of total Zn (75 µg/L) for direct stormwater discharges in Sweden (Table S2). With regard to dissolved Zn concentrations associated with exposure galvanized steel pipes, concentrations of up to 1400 µg/L were determined in semi-synthetic stormwater and up to 670 µg/L in synthetic stormwater. Whilst the dissolved and bioavailable concentrations are not directly inter-changeable (Lindfors et al. 2021), determined dissolved concentrations are up to two orders of magnitude higher than the UK bioavailable EQS standard for Zn (i.e. 10.9 µg/L in surface waters; Table S2). Only buffered waters were also reported to leach Pb from galvanized corrugated steel pipes and to leach Cu and Zn from PVC pipes at concentrations which exceed the Swedish pertinent stormwater discharge guideline values (8 µg/L for total Pb and 18 µg/L for total Cu) (Table S2). Reported dissolved Cu concentrations in semisynthetic and field-collected stormwater after exposure to concrete, PVC, and galvanized corrugated steel pipes were again in excess of the UK bioavailable EQS for Cu (1 µg/L) and can be harmful to receiving water biota.

Only a limited number of studies have evaluated the impact of pipe rehabilitation technologies on stormwater quality. However, the studies that are available indicate that SIPP and CIPP applications can result in the release of several organic substances (e.g. styrene, vinyl, benzaldehyde, DEP, and DBP), with the source likely to be their release from resin during and after installation processes. Whilst the level of release of organic substances (e.g. styrene) is less for UV-cured CIPP than for thermal-cured CIPP, styrene has been detected at concentrations identified as toxic to aquatic organisms under both processes. The exact composition of CIPP condensate is unknown making understanding of its environmental impacts of it difficult to predict. Overall, the concentration of organic contaminants releasing from pipe liners may vary with the type of CIPP material used, its curing method (UV or thermal), the water type, and site conditions (i.e. high or intermittent water flow). The potential for organic contaminant release has been recognized by NASSCO (trade association for trenchless technologies) who have developed specific guidance to support the safe handling of resin materials, prevention of particulate emissions to air and water during CIPP cutting processes, and the proper disposal of thermal or steam condensate and extruded resin cuttings. However, the extent to which such best practice is implemented in the field is not clear as installation activities are not routinely monitored to determine levels of compliance.

The contributions of different pipe materials and pipe repairs (SIPP/CIPP) to stormwater quality changes have only been examined to a limited extent in terms of (i) the use of field-collected stormwater, (ii) in-transport changes in total and dissolved metals content and organic substances, and (iii) change in impacts as pipes age over time. Future studies are therefore needed to provide a better understanding of the organic and inorganic substances contributed to stormwater by commonly used pipe materials and repair techniques. Moreover, incomplete curing of resin during CIPP/poor condensate management may result in contaminant release and levels of compliance with best practice need further investigation. Based on this review, the use of flowing water experimental design techniques and field-collected (as opposed to synthetic) stormwaters is recommended in such studies. Furthermore, future work could include field monitoring studies of CIPP and SIPP applications to understand which chemicals are released into the environment during and post installation (e.g. water samples should be screened for wider range of substances) with eco-toxicity tests undertaken to enable a complete assessment of their impacts on aquatic species to be derived.

### Supplementary Information

Below is the link to the electronic supplementary material.

Supplementary file1 (DOCX 173 KB)

## Data Availability

The data extracted from literature (selected for this review) is presented in the paper and supplementary file in the form of tables and figures.
