# Supplementary material for: Impacts of stormwater pipe materials and pipe repairs on stormwater quality: a review
Source: Environ Sci Pollut Res Int. 2023 Nov 4;30(56):118065–77. doi: 10.1007/s11356-023-30508-6 (PMC10697894; doi:10.1007/s11356-023-30508-6)
Supplement: Supplementary file 1 — Supplementary file1 (DOCX 173 KB) [file 11356_2023_30508_MOESM1_ESM.docx]

Supplementary file

**Impacts of stormwater pipe materials and pipe repairs on stormwater quality: A review**

Mehwish Taneez, Heléne Österlund, Lian Lundy, Maria Viklander

Urban Water Engineering, Department of Civil, Environmental and Natural Resources Engineering, Luleå University of Technology, 971 87, Luleå, Sweden

**Table S1.** Pipe materials, experimental conditions and parameters analysed

| **Authors** | **Pipe materials evaluated** | **Water source** | **Flowing water or pipe immersed** | **Pipe length** | **Flow rate and exposure time** | **Parameters analysed** |
| --- | --- | --- | --- | --- | --- | --- |
| Perkins et al. 2005 | PVC and concrete | Synthetic stormwater-Cu spiked (2.391 mg/L) | Flowing (using a pump) | 6100 mm | 0.325 L/min, 0.75 L/min, 1.5 L/min; samples taken after 10 and 75 mins | Total and dissolved Cu |
| Davies et al. 2010a | Steel and fiber reinforced concrete, old concrete, and PVC | Roof runoff | Flowing (using a pump) | 1600 mm (new pipes); 1800 and 800 mm (old pipes) | 0.5 L/s for 120 minutes (samples taken after 5, 10, 20, 30, 40, 60, 90 and 120 mins) | pH, EC, total alkalinity (as bicarbonate), Ca, total anions and cations |
| Davies et al. 2010b | Concrete and PVC | Roof runoff, urban creek and non-urban creek | Flowing (manually) | 1400 mm | 20 L of water at rate of 0.2 L/s for 100 min (samples taken after 0, 25, 50, 75, 100 mins) | pH, EC, total alkalinity (as bicarbonate), Cl, Ca, Na, K, Mg, SO_4_, total anions and cations |
| Ogburn et al. 2013 | Concrete, PVC, HDPE, and GCS | Parking lot runoff adjusted to pH 5 or 8 using buffer chemicals disodium phosphate dehydrate Na_2_HPO_4_4. 2H_2_O and potassium phosphate monobasic KH_2_PO_4_, bay and river water | Pipe sections immersed; concrete in 180 L buckets while other pipes in 16 L buckets | 153 mm (concrete)  305 mm (all other pipes) | 16 - 180 L immersion volume for 0.5 h, 1 h, 27 h, 1 week, 1 month, 2 and 3 months | pH, EC, total alkalinity, Cl, Ca, Cu, Pb and Zn |
| Grella et al. 2016 | Steel reinforced concrete and epoxy treated concrete | Roof runoff | Flowing (using a pump) | 1600 mm | 0.25 L/s for 100 minutes | pH, EC, total bicarbonate, Na, Ca, K, total anions and cations |
| Borris et al. 2017 | Concrete, PVC, GCS | Synthetic, semi-synthetic and field collected stormwater | Flowing (using a pump) | 500 mm | 0.95 L/min for 20 min, four 20 min cycles (first run discarded) | pH, turbidity, particle size distribution, total and dissolved Cu, Pb, Zn |
| Purdy et al. 2021 | Concrete and PVC | Upland swamp water | Flowing (using a pump) | 2500 mm | 0.44 L/s for 60 min | pH, EC, Na, Ca, K, Mg, Al, As, Ba, Cr, Pb and Sr |
| Donaldson 2009 | CIPP (Feltliner-styrene based, steam/hot water cured) | Stream/creek water samples were collected from 6 sites (1 m to 40 m downstream) and from 1 site tap water was poured at pipe inlet and collected from the outlet | Flowing | Field installation, pipe length varied between sites i.e., from 60 ft (18288 mm) – 121 ft (36880 mm) | 30 to 116 days  4 sites; continual water flow, 3 site; intermittent water flow | Styrene |
| Ren and Smith 2012 | Ultraliner (PVC alloy) and troliner (HDPE based and has specially designed embed studs on its outer surface)) | De-ionised water | Immersed (batch tests) | 5 g of each liner was placed in 15-20 ml tube containing deionized water | Samples taken immediately and after 6, 12, 24 and 48 h | Bisphenol A (BPA), di-(2-ethylhexyl) phthalate (DEHP), and benzyl butyl phthalate (BBP) |
| Whelton et al. 2013 | Spray on liners: Polymer enhanced cement mortar (PECM) and poly (etherurethene) urea (PEUU) | Synthetic water | Immersed sections of pipe liners | surface area to  water volume ratio PECM = 0.16 cm^2^/mL; PEUU=1.04 cm^2^/mL | Samples collected and immersion water replaced every 3 days for 30 days | pH, alkalinity, TOC, DOC, and TN |
| Donaldson and Whelton 2013 | SIPP-cementitious and polyurea  CIPP (Feltliner- styrene based, steam/hot water cured)  CIPP (Feltliner- vinyl-ester based styrene free resin, steam/hot water cured)  CIPP (Fiber glass- vinly ester styrene based, UV cured) | Tap water, synthetic water | Both | Immersed in tap water: UV-CIPP the surface area of liner to volume ratio was 70:1  Immersed in synthetic water: PECM = 0.16 cm^2^/mL; PEUU=1.04 cm^2^/mL  Flowing water: pipe length for SIPP; 20 ft (6096 mm),  Steam-CIPP; 60 ft (18288 mm), UV-CIPP; 35 ft (10668 mm) | SIPP: samples were taken after 2 and 10 day (flowing water) and 30 days (immersion)  Steam- CIPP:  the samples were analysed after 30 min of installation to 120 days (flowing water; flow rate not given)  UV-CIPP: the samples were analysed after 30 min to 49 days (flowing water) and 1 day to 49 days (immersion) | pH, vinyl, acrylate, styrene, methylene diphenyl diiocyanate (MDI), methyenedianiline (MDA), TOC, COD, and TN |
| Tabor et al. 2014 | CIPP (Feltliner, steam/hot water cured) | Stormwater samples collected at culvert inlet, outlet, and downstream | Flowing | Field installation  Pipe length: site 1; 158 ft (48158 mm), site 2; 235 ft (71628 mm) | Samples collected 0, 7, 28, 35 days (flow rate not given) | Styrene, TOC, DOC, Al, Ba, Ca, Cu, Pb and Zn |
| Li et al. 2019 | CIPP (Fiber glass, UV cured) | Tap water | Flowing | Field installation  Pipe length: site 1 and 2; 30.5 m (30500 mm), site 3; 15.2 (15200 mm), site 1 (other location); 26.5 m (26500 mm) | Samples were collected after installation (flow rate not given) | pH, Styrene, phenols, dibutyl phthalate |

Key: min = minutes, mm= millimeter, ft= feet, PVC= polyvinyl chloride, HDPE= high density polyethylene, GCS= galvanized corrugated steel, SIPP= spray in place pipe, CIPP= cured in place pipe, total organic carbon (TOC), dissolved organic carbon (COD) and total nitrogen (TN)


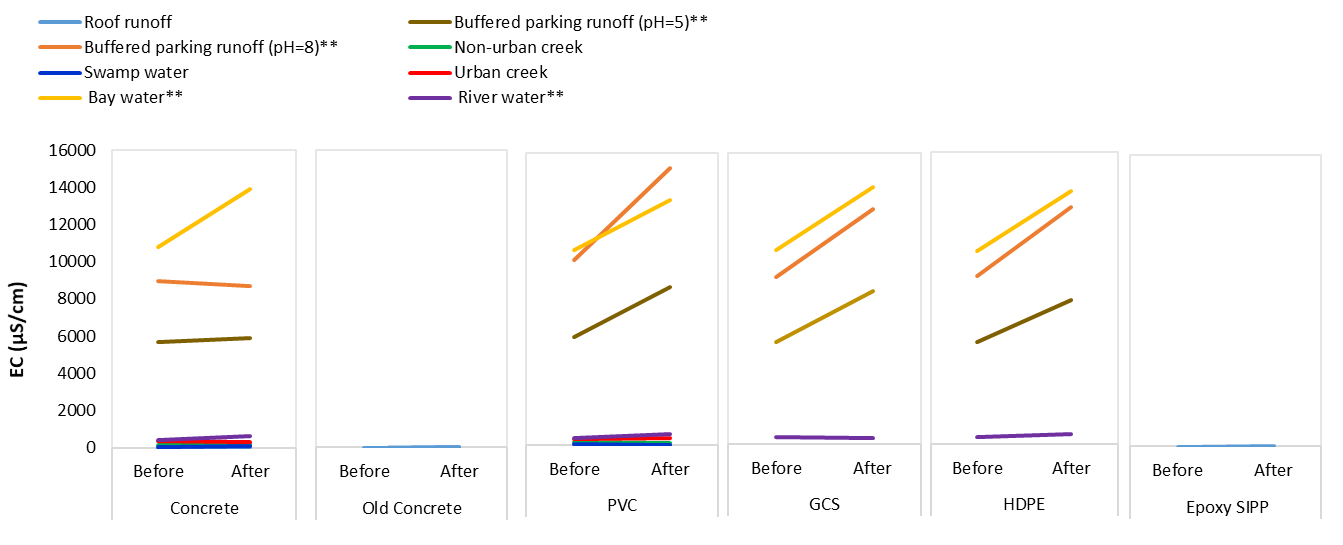


**Figure S1.** Before and after EC values of various water types following contact with pipe materials in flowing and immersion (**) studies, data adapted from (Davies et al. 2010c, b; Ogburn et al. 2013; Grella et al. 2016; Purdy et al. 2021)

Key: PVC= polyvinyl chloride, GCS= galvanized corrugated steel, HDPE= high density polyethylene and SIPP= spray in place pipe


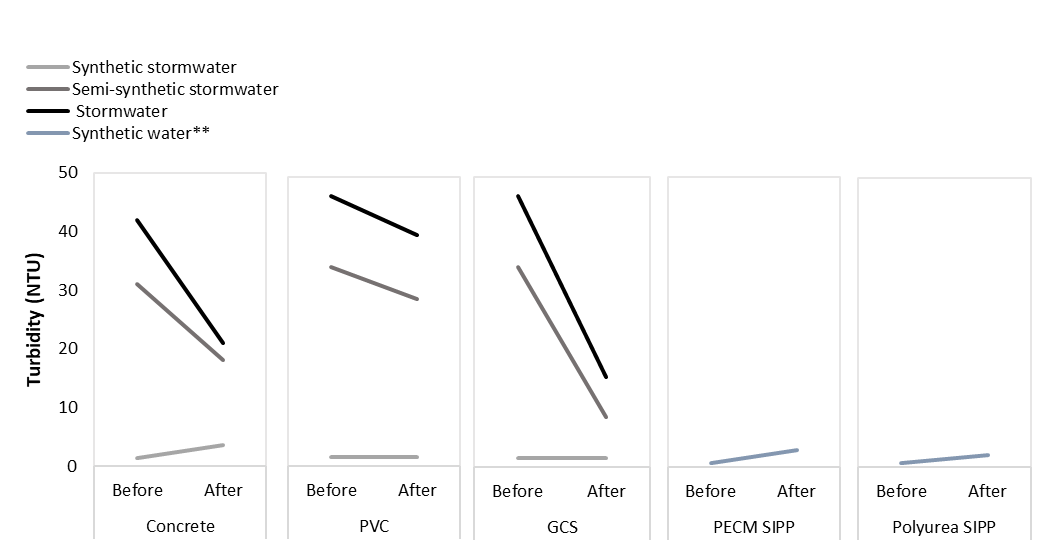


**Figure S2.** Turbidity changes in contact waters on exposure to pipe materials in flowing and immersion (**) studies, data adapted from (Whelton et al. 2013; Borris et al. 2017)

Key: PVC= polyvinyl chloride, GCS= galvanized corrugated steel, PECM= polymer enhanced cement mortar and SIPP= spray in place pipe


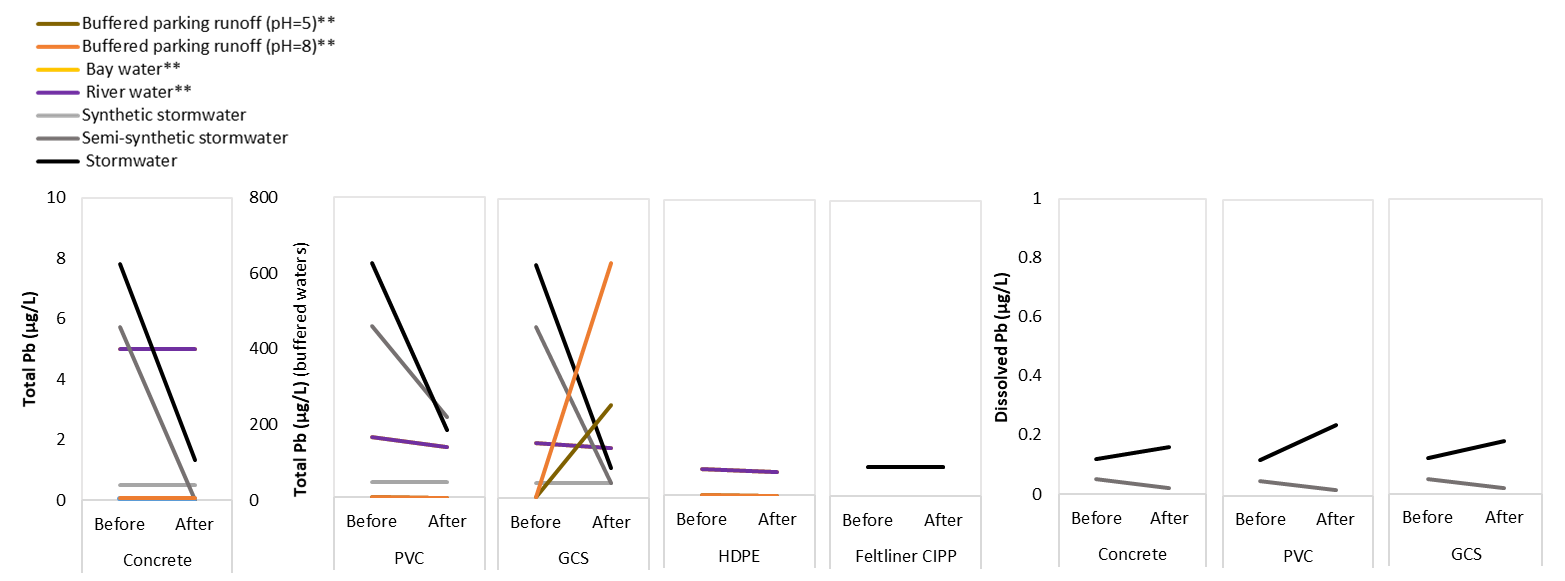


**Figure S3.** Total and dissolved Pb concentrations in contact waters exposed to pipe materials in flowing and immersion (**) studies, data adapted from (Ogburn et al. 2013; Borris et al. 2017)

Key: PVC= polyvinyl chloride, GCS= galvanized corrugated steel, HDPE= high density polyethylene and CIPP= cured in place pipe

**Table S2.** Suggested guideline values for stormwater discharge quality and environmental quality standards (EQS) for surface waters (total, dissolved and bioavailable fraction of metals)

|  | Suggested guideline values for  direct stormwater discharge in Sweden | EQS for surface waters | |
| --- | --- | --- | --- |
| Source | (Alm et al. 2010) | EU AA-EQS  (EC directive 2013/39 2013) | UK AA-EQS  (UK TAG 2013) |
| Metal fraction (µg/L) | Total metals | Dissolved metals (<0.45µm) |  |
| Cu | 18 |  | 1 |
| Pb | 8 | 1.2 |  |
| Zn | 75 |  | 10.9 |

**References**

Alm H, Banach A, Larm T (2010) Occurrence and Treatment of Priority Substances, Metals and Other Substances in Stormwater (Förekomst Och Rening Av Prioriterade Ämnen, Metaller Samt Vissa Övriga Ämnen i Dagvatten). SVU, Stockholm

Borris M, Österlund H, Marsalek J, Viklander M (2017) An exploratory study of the effects of stormwater pipeline materials on transported stormwater quality. Water Sci Technol 76:247–255. https://doi.org/10.2166/wst.2017.195

Davies PJ, Wright IA, Findlay S, Jonasson O (2010a) The effect of the in-transport process on urban water chemistry an examination of the contribution of concrete pipes and gutters on urban water quality. NOVATECH 2010-Proceedings 7th Int Conf Sustain Tech Strateg Urban Water Manag 1–10

Davies PJ, Wright IA, Jonasson OJ, Findlay & SJ (2010b) Impact of concrete and PVC pipes on urban water chemistry. Urban Water J 7:233–241. https://doi.org/10.1080/1573062X.2010.484502

Donaldson BM (2009) Environmental Implications of Cured-in-Place Pipe Rehabilitation Technology. Transp Res Rec 2123:172–179. https://doi.org/10.3141/2123-19

Donaldson BM, Whelton AJ (2013) Impact of Stormwater Pipe Lining Materials on Water Quality: Field Study and Resulting Specifications. Transp Res Rec 2362:49–56. https://doi.org/10.3141/2362-07

EC directive 2013/39 (2013) Directive 2013/39/EU of the European Parliament and of the Council of 12 August 2013 amending Directives 2000/60/EC and 2008/105/EC as regards priority substances in the field of water policy. Off J Eur Union L 226:1–17

Grella C, Wright IA, Findlay SJ, Jonasson OJ (2016) Geochemical contamination of urban water by concrete stormwater infrastructure: applying an epoxy resin coating as a control treatment. Urban Water J 13:212–219. https://doi.org/10.1080/1573062X.2014.951660

Li X, Ra K, Nuruddin M, et al (2019) Outdoor manufacture of UV-Cured plastic linings for storm water culvert repair: Chemical emissions and residual. Environ Pollut 245:1031–1040. https://doi.org/10.1016/J.ENVPOL.2018.10.080

Ogburn O, Pitt RE, Clark S (2013) The effects of water quality characteristics on pollutant releases from drainage materials. J Water Manag Model 235–266

Perkins C, Nadim F, Arnold WR (2005) Effects of PVC, cast iron and concrete conduit on concentrations of copper in stormwater. Urban Water J 2:183–191. https://doi.org/10.1080/15730620500236658

Purdy K, Reynolds JK, Wright IA (2021) Potential water pollution from recycled concrete aggregate material. Mar Freshw Res 72:58–65

Ren DE, Smith JA (2012) Evaluation of Environmental Impacts of Two Common Restoration Methodologies for Pipes that Convey Stormwater Runoff. Bull Environ Contam Toxicol 89:557–562. https://doi.org/10.1007/s00128-012-0742-4

Tabor ML, Newman D, Whelton AJ (2014) Stormwater Chemical Contamination Caused by Cured-in-Place Pipe (CIPP) Infrastructure Rehabilitation Activities. Environ Sci Technol 48:10938–10947. https://doi.org/10.1021/es5018637

UK TAG (2013) UK TAG Environmental Standards Phase 3 Final Report. United Kingdom Technical Advisory Group on the Water Framework Directive.

Whelton AJ, Maryam S, Matthew T, et al (2013) Impact of Infrastructure Coating Materials on Storm-Water Quality: Review and Experimental Study. J Environ Eng 139:746–756. https://doi.org/10.1061/(ASCE)EE.1943-7870.0000662
